# Supplementary material for: Segmentation strategy of de novo designed four-helical bundles expands protein oligomerization modalities for cell regulation
Source: Nat Commun. 2023 Apr 8;14:1995. doi: 10.1038/s41467-023-37765-6 (PMC10082849; doi:10.1038/s41467-023-37765-6)
Supplement: Supplementary file 1 — Supplementary Information [file 41467_2023_37765_MOESM1_ESM.pdf]

SUPPLEMENTARY INFORMATION

Supplementary Figure 1. – 12.

Supplementary Table 1: Statistical analysis of rapamycin inducible 1:3 dimerization.

Additional supplementary materials include:

Supplementary Data 1: amino acid sequences of constructs used in this study.

SUPPLEMENTARY FIGURES

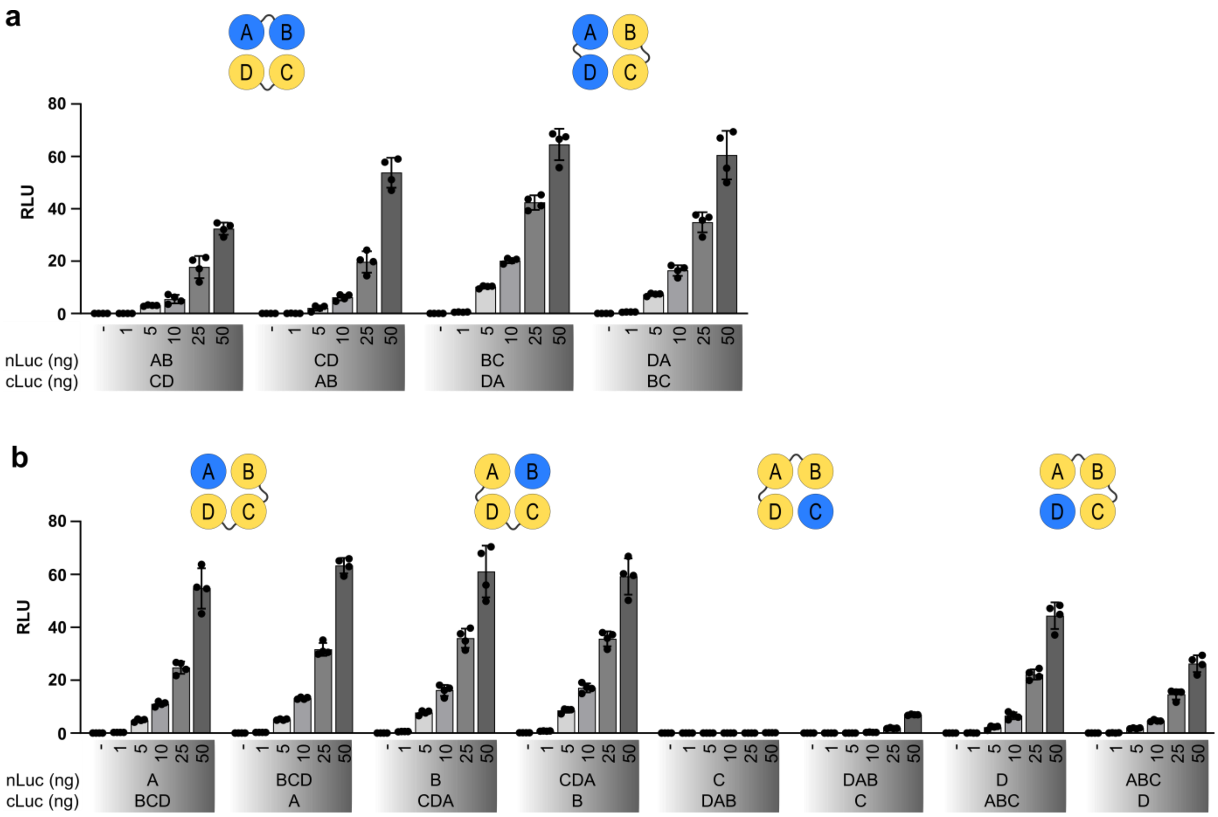

Supplementary Fig. 1. Titrations of dimerization segmentation domains of split4HB in HEK293T cells. a-b, Titration of all possible nLuc and cLuc fusion combinations of 2:2 dimerization domains (**a**) and 1:3 dimerization domains (**b**). Values are the mean of four biological replicates ± (s.d.) and representative of three independent experiments.

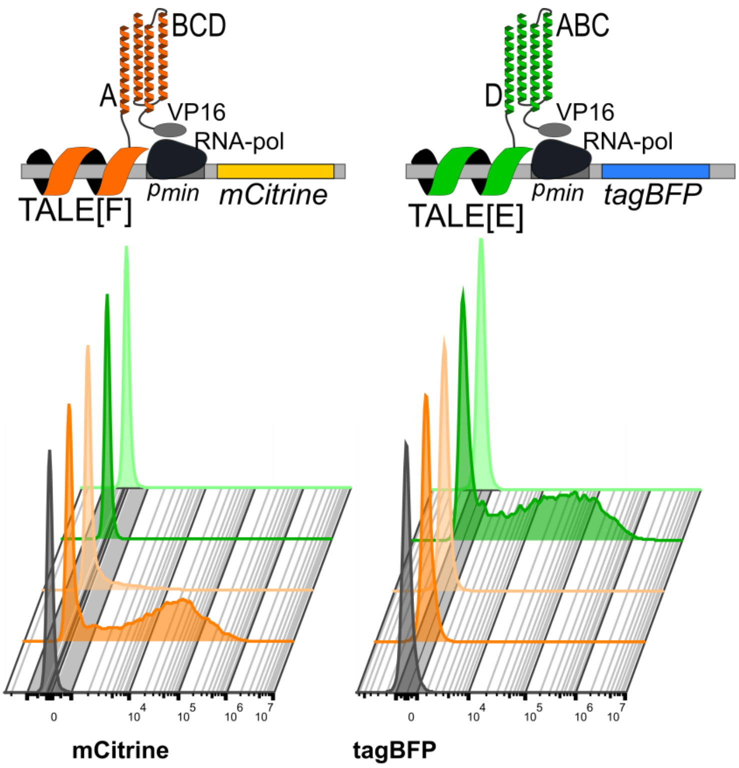

	10F_pmin mCitrine	10E_pmin tagBFP	TALE[F] **A**	**BCD** VP16	TALE[E] **D**	**ABC** VP16
(light green)	+	+	-	+	+	-
(green)	+	+	-	-	+	+
(light orange)	+	+	+	-	-	+
(orange)	+	+	+	+	-	-
(grey)	+	+	-	-	-	-

Supplementary Fig. 2. Simultaneous application of two 1:3 dimerization modules. Flow cytometry evaluation of cross-talk between parts of two segmentation modules A:BCD and D:ABC in HEK293T. HEK293T cells were transiently transfected with combination of TALE reporter plasmids, DNA binding TALE fused to one segment of 4HB and transcriptional activators fused to counterparts of 4HB dimerization modules as noted in the legend. The dimerization of A:BCD results in mCitrine expression. The dimerization of D:ABC results in tagBFP expression. Graphs are representative of two independent experiments.

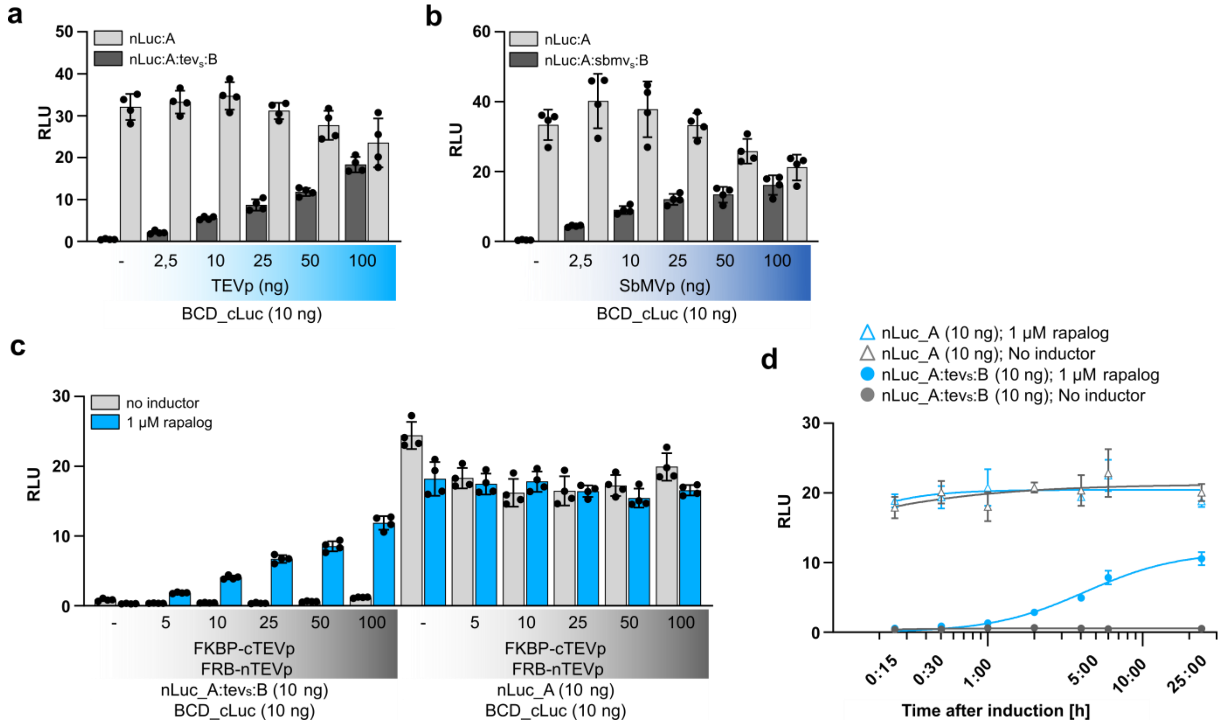

Supplementary Fig. 3. Determination of protease amount effects on inducible and non-inducible split4HB dimerization. a-b, Evaluation of TEVp (**a**) and SbMVp (**b**) amount on non-inducible (light gray columns) and inducible dimerization (dark gray columns) of split4HB. HEK293T cells were transiently transfected with 10 ng of plasmids for non-inducible, TEVp-inducible (**a**) or SbMVp-inducible (**b**) split4HB dimerization domains and increasing amounts of plasmid encoding for constitutively expressed TEVp (**a**) or SbMVp (**b**). **c**, Evaluation of splitTEVp amount on inducible (left) and non-inducible (right) dimerization of split4HB in the absence (gray columns) and presence (blue columns) of 1 μM rapalog. HEK293T cells were transiently transfected with 10 ng of plasmids for non-inducible or TEVp inducible split4HB dimerization domains and increasing amounts of plasmids encoding for splitTEVp on rapalog inducible FRB and FKBP domains. **d**, Kinetics of exogenously regulated split4HB dimerization domains in the absence (gray circles) and presence of 1 μM rapalog (blue circles). Comparison with non-inducible split4HB dimerization in the absence (gray open triangles) and presence of 1 μM rapalog (blue open triangles). HEK293T cells were transiently transfected with 10 ng of plasmids for split4HB dimerization domains (nLuc_A:tevs:B or nLuc_A and BCD_cLuc). HEK293T cells were co-transfected with 10 ng of plasmids for split4HB dimerization domains and 50 ng of plasmids for FKBP-cTEVp and FRB-nTEVp constructs. Values in **a-c** are the mean of four biological replicates ± (s.d.) and representative of three independent

experiments. Values in **d** are single data points of four biological replicates and representative of three independent experiments.

Supplementary Table 1: Statistical analysis of rapamycin inducible 1:3 dimerization.

time from induction	0:15:00	0:30:00	1:00:00
Column A	nLucA:TEVsB, BCDcLuc<br>no inductor		
vs.	vs.		
Column B	nLucA:TEVsB, BCDcLuc<br>1uM rapamycin		
Unpaired t test			
P value	0.0058	0.000144	<0.0001
P value summary	**	***	****
Significantly different (P < 0.05)?	Yes	Yes	Yes
One- or two-tailed P value?	Two-tailed	Two-tailed	Two-tailed
t, df	t=4.178, df=6	t=8.511, df=6	t=15.02, df=6

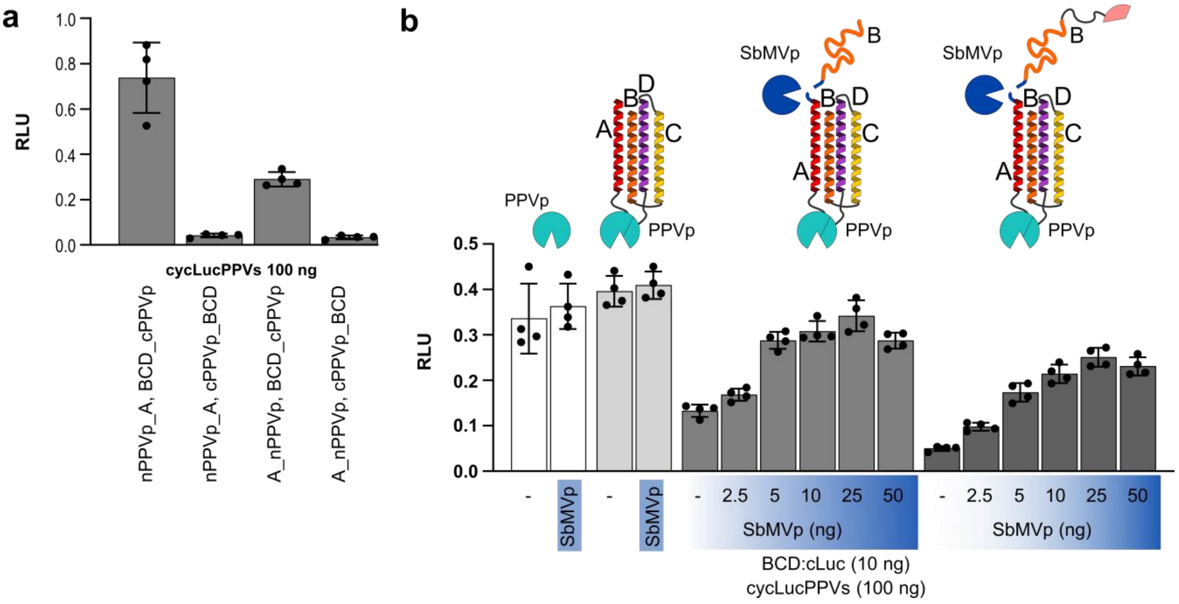

Supplementary Fig. 4. Further characterization of splitPPVp and inducible SbMVp-PPVp protease cascade. a, SplitPPVp reconstitution efficiency of N- and C-terminal fusion combinations on split4HB dimerization domains. **b**, Assessing inducible SbMVp-PPVp protease cascade efficiency. Left to right: constitutively expressed PPVp in absence and presence of SbMVp; splitPPVp in fusion with non-inducible split4HB dimerization domains in absence and presence of SbMVp; splitPPVp in fusion with SbMVp inducible split4HB dimerization domains with inhibitory peptide B in absence and presence of increasing amounts of SbMVp; splitPPVp in fusion with SbMVp inducible split4HB dimerization domains with inhibitory peptide B and inactive cPPVp* domain in abscence and presence of increasing amounts of SbMVp. HEK293T cells were transiently transfected with 100 ng cycLuc_ppvs plasmid, 2.5 ng plasmid encoding for non-inducible or inducible split4HB dimerization domains in fusion with splitPPVp domains and variable amount of SbMVp plasmid. Values are the mean of four biological replicates ± (s.d.) and representative of three independent experiments.

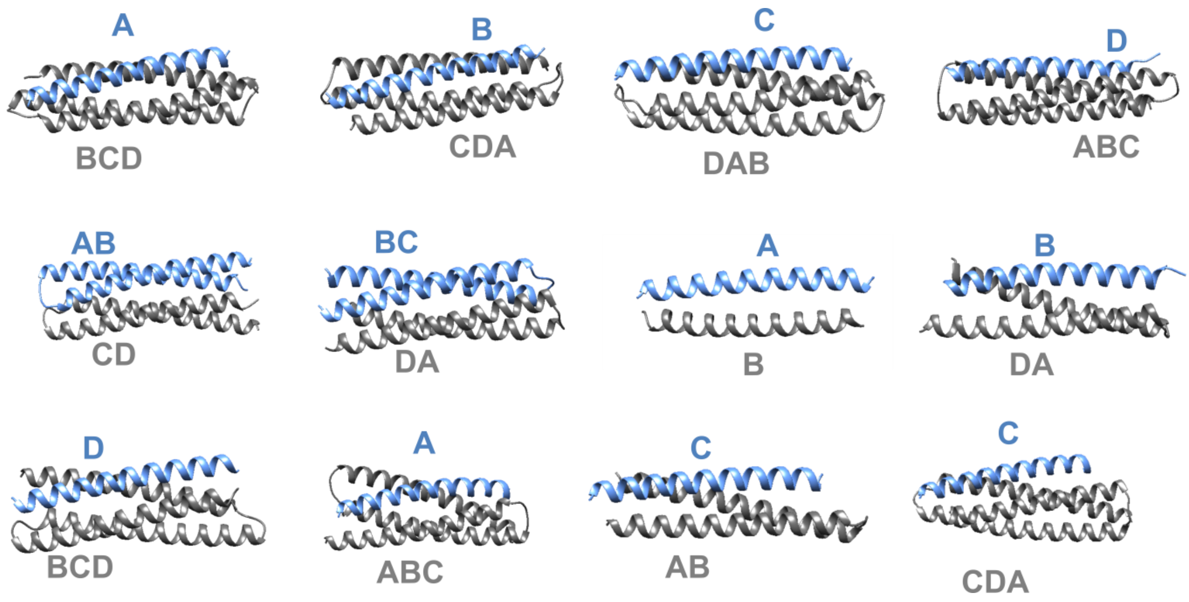

Supplementary Fig. 5. Analysis of 4HB segmentations with protein structure prediction tool AlphaFold2 modeling. AlphaFold2 modeling tool returns helical protein structure predictions in all cases, regardless of number of input helices and their affinity.

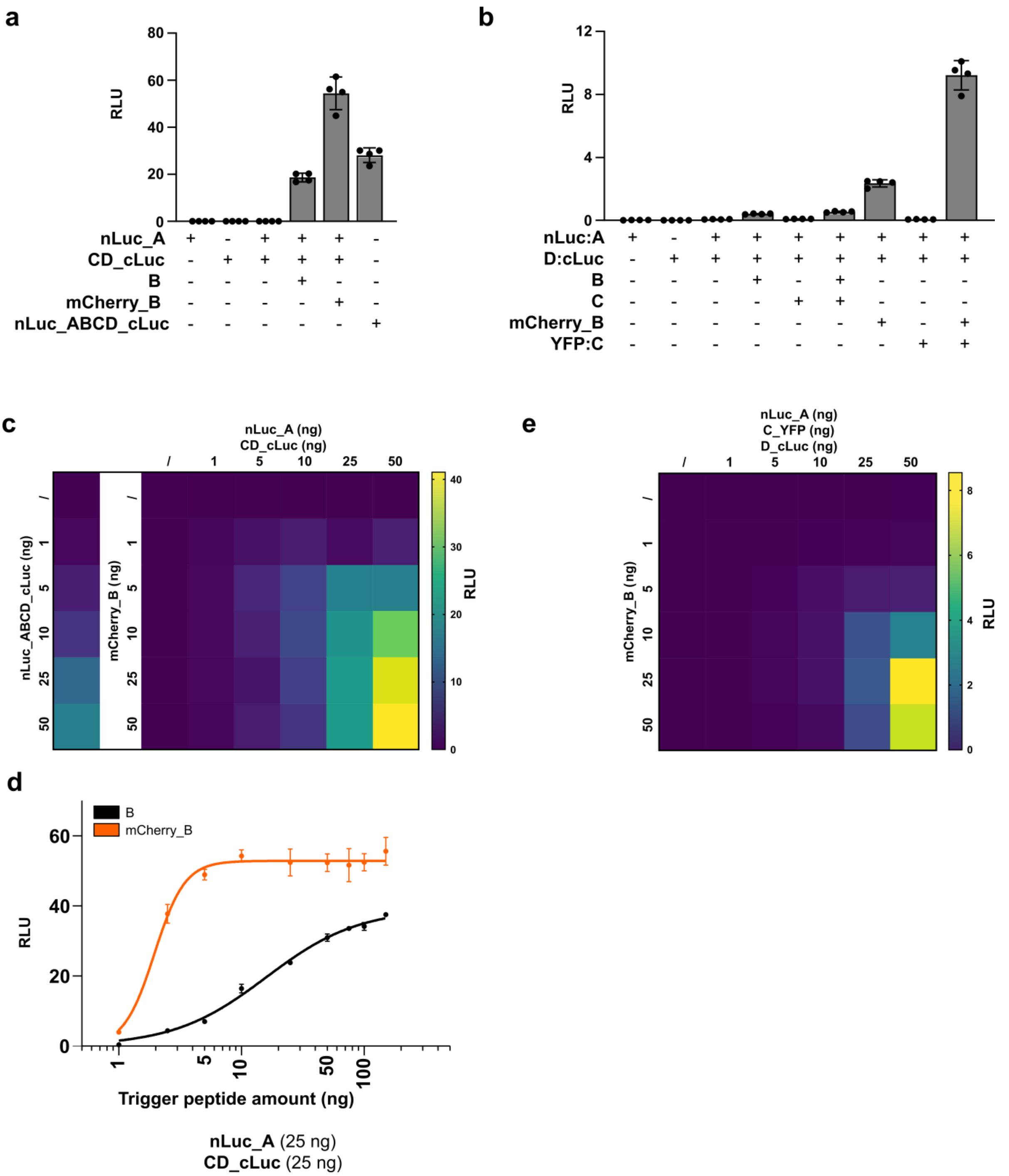

Supplementary Fig. 6. Fusion of fluorescent proteins to single peptide modules enhances their stability enough to promote trimerization and tetramerization of split4HB. a-b, Reconstitution efficiency of split4HB oligomerization domains on luciferase reporter. Single peptide modules B in trimerization (**a**) or B and C in tetramerization (**b**) were used on their own or in fusion with fluorescent proteins mCherry and YFP, respectively. **c**, Concentration dependence of trimerization domains on luciferase reporter system. mCherry_B was titrated independently of other constructs. **d**, Trimerization

reconstitution dependence on amount of “trigger“ peptides without or with fusion protein mCherry. Experimental values were fitted as nonlinear regression using Allosteric sigmoidal Least squares fit equation Y=Vmax*X^h/(Khalf^h+ X^h). **e**, Concentration dependence of tetramerization domains on luciferase reporter system. mCherry_B was titrated independently of other constructs. Values in (**a-b** and **d**) are the mean of four biological replicates ± (s.d.) and representative of three independent experiments. Values in (**c** and **e**) are the mean of four biological replicates and representative of three independent experiments.

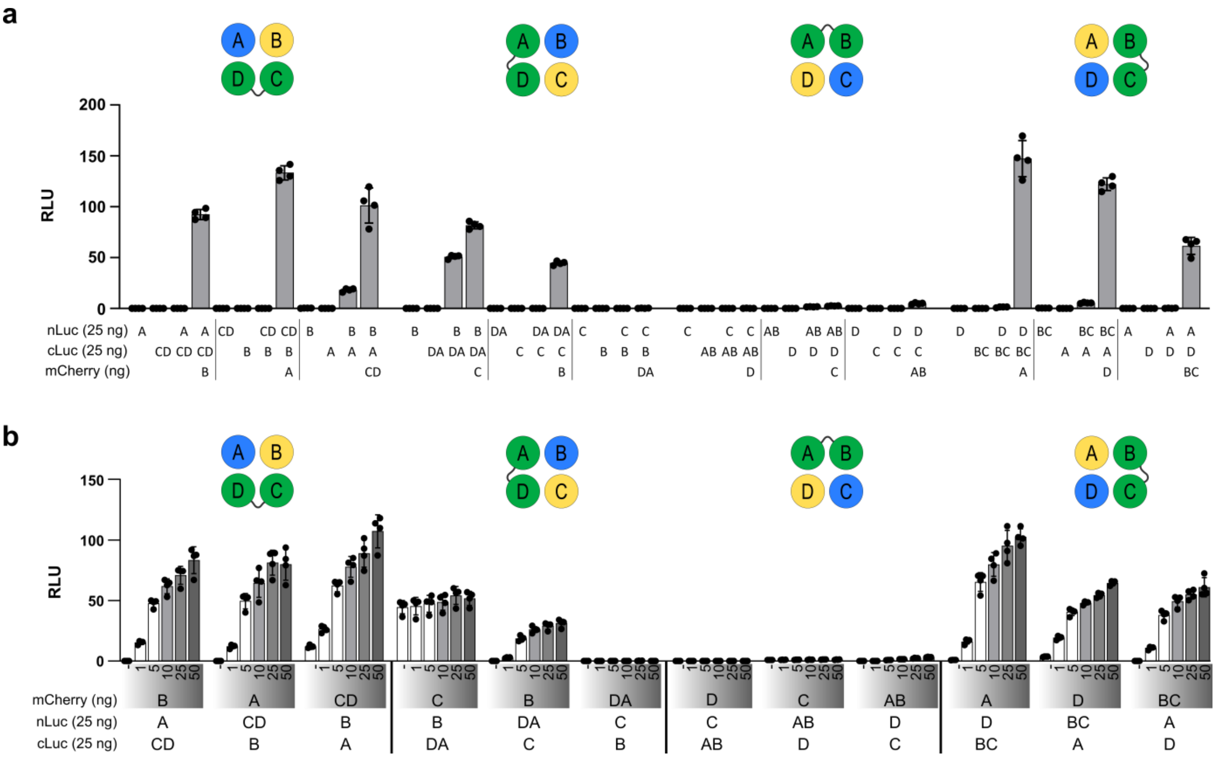

Supplementary Fig. 7. Titrations of trimerization segmentation domain of split4HB in HEK293T cells. a, Determining background reconstitution signals in all possible trimerization combinations. **b**, Titration of „trigger“ peptides in all possible trimerization combinations. Values are the mean of four biological replicates ± (s.d.) and representative of three independent experiments.

a

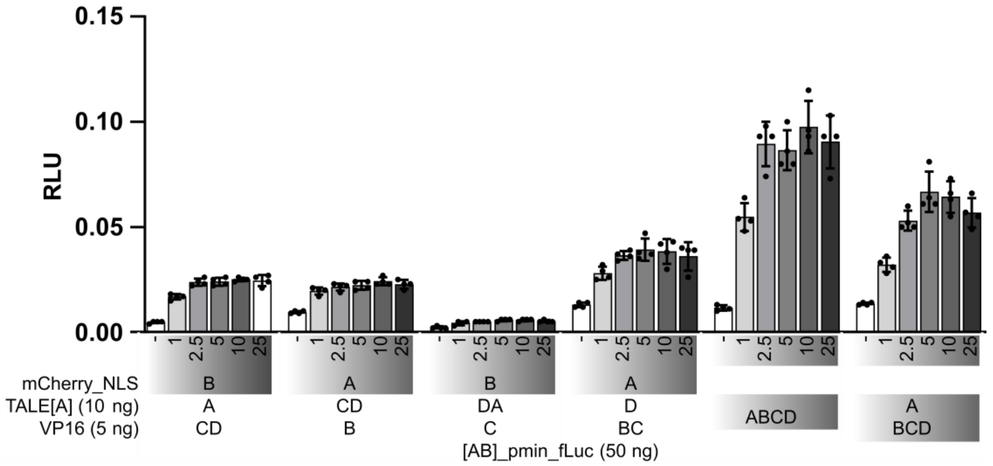

b

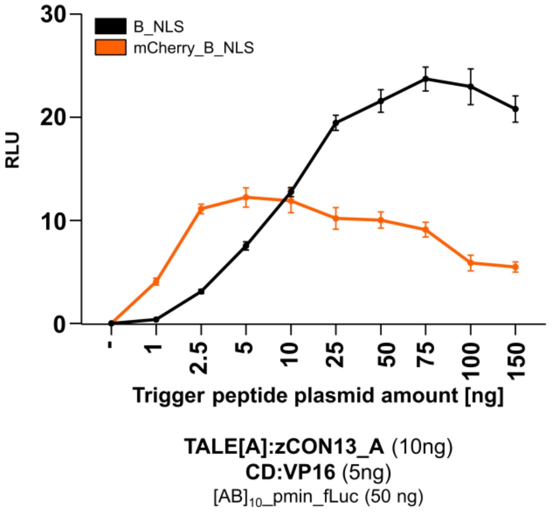

Supplementary Fig. 8. Trimerization of split4HB split domain is highly effective for transcription regulation. a, Determining trimerization efficiency from different split4HB trimerization domains. HEK293T cells were transiently transfected with 50 ng 1AB_pmin_fLuc plasmid, 10 ng of plasmid encoding one of the trimerization domains in fusion with TALE[A], 5 ng of plasmid encoding second trimerization domain in fusion with VP16 and various amounts of „trigger“ peptide X_NLS in fusion with mCherry. **b**, Trimerization reconstitution dependence on amount of „trigger“ peptides without or with fusion protein mCherry. Values are the mean of four biological replicates ± (s.d.) and representative of three independent experiments.

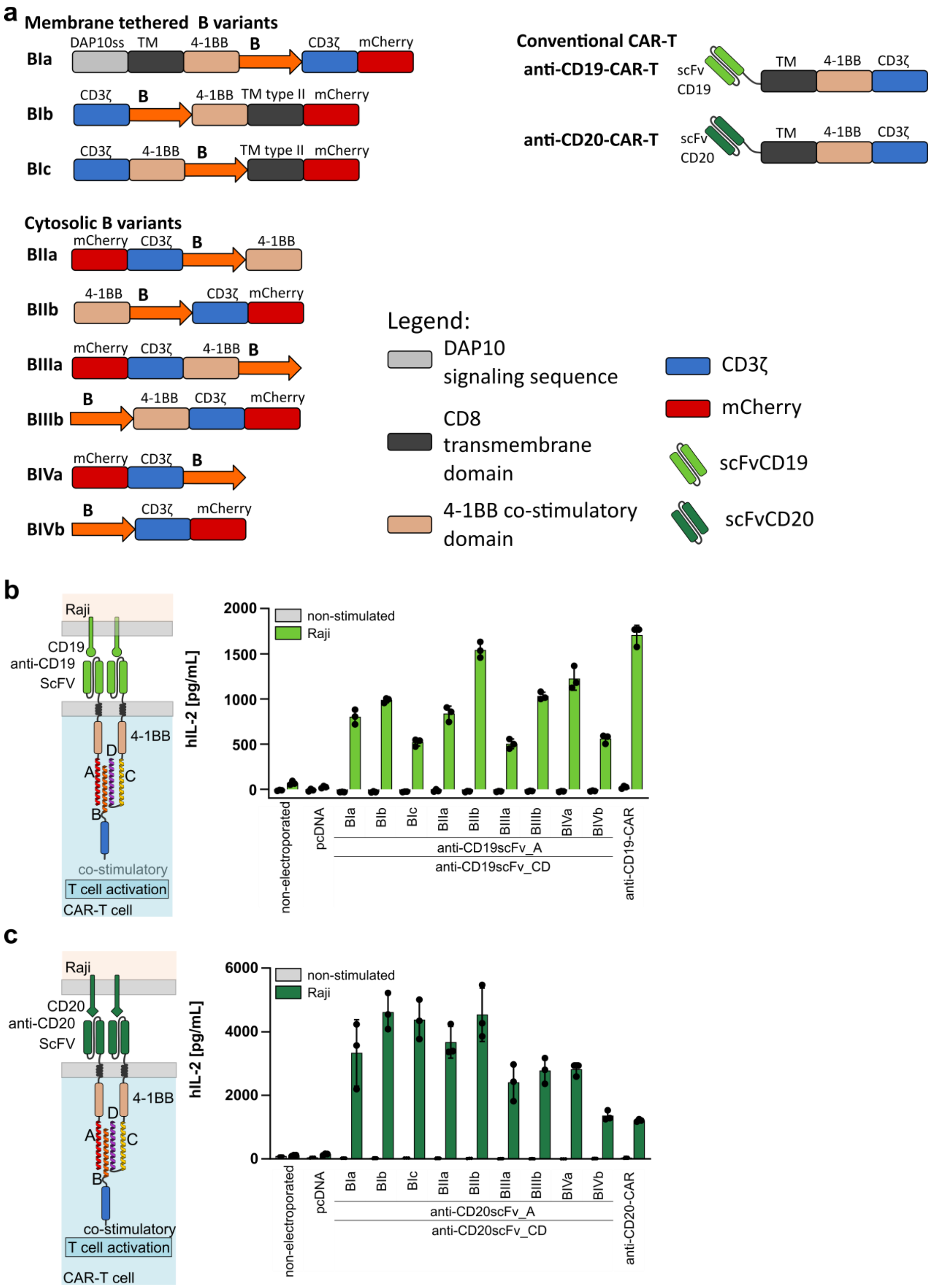

Supplementary Fig. 9. Evaluation of trimerization domains for use in designed CAR-T cells against CD19 or CD20. a, Schematic of peptide B in various fusion configurations with CD3ζ activator domain and 4-1BB co-stimulatory domain and conventional CAR-T constructs. **b-c**, Analysis of IL-2 production of split4HB trimerization domains in anti-CD19 CAR-T (**b**) and anti-CD20 CAR-T (**c**) systems post co-cultivation with double positive ($CD19^{+}/CD20^{+}$) target Raji cells in E:T ratio of

10:1. Values are the mean of three biological replicates ± (s.d.) and representative of two independent experiments. E: effector cells; T: target cells; scFVCD19: single chain fragment variable, antibody against CD19 ligand; scFVCD20: single chain fragment variable, antibody against CD20 ligand.

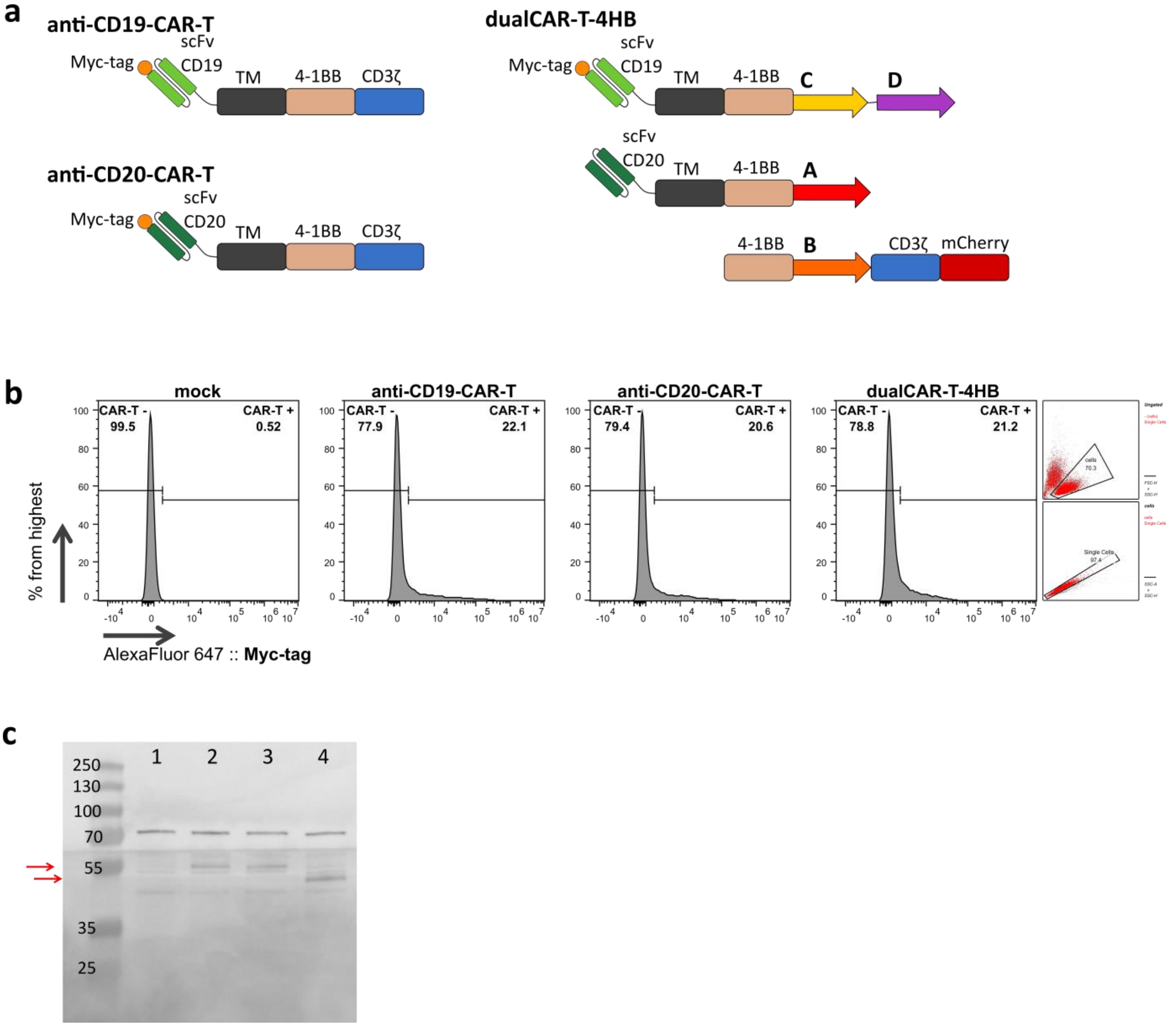

Supplementary Fig. 10. Comparison of expression of CAR constructs. **a**, Schematic of 2nd generation antiCD19-CAR, antiCD20-CAR and dualCAR-T-4HB showing N-terminal Myc-tag. Jurkat cells were electroporated with 5 ug of construct(s) using Neon electroporation system. **b**, Flow cytometry analysis of CAR construct expression. 48h after electroporation 5×10^5 cells were collected and dyed with antiMyc-tag:AlexaFluor647 antibodies at 1:100 ratio. Presented is the comparison between (from left to right) empty pcDNA3 vector electroporated Jurkat cells (mock) 2nd generation antiCD19-CAR, 2nd generation antiCD20-CAR and dualCAR-T-4HB with presented gating strategy. **c**, Western blot was performed from Jurkat cell, collected 48 h post electroporation. Cell lysates were prepared using Ripa buffer, supplemented with peptide inhibitors. Detection of Myc-tagged CAR constructs was performed using primary rabbit-antiMyc-tag antibodies at 1:2000 and secondary antibodies Goat anti-rabbit:HRP at 1:3000 ratio. Samples are as follows: (from left to right) empty

pcDNA3 vector electroporated Jurkat cells (**1**), 2nd generation antiCD19-CAR (**2**), 2nd generation antiCD20-CAR (**3**) and dualCAR-T-4HB (**4**). Observed specific bands are accentuated with red arrows, at 55 kDa for 2nd generation CAR constructs, 46,8kDa for dualCAR-T-4HB constructs and 70kDa for loading control Hsp70. Presented data in **b** and **c** are representative of two independent experiments.

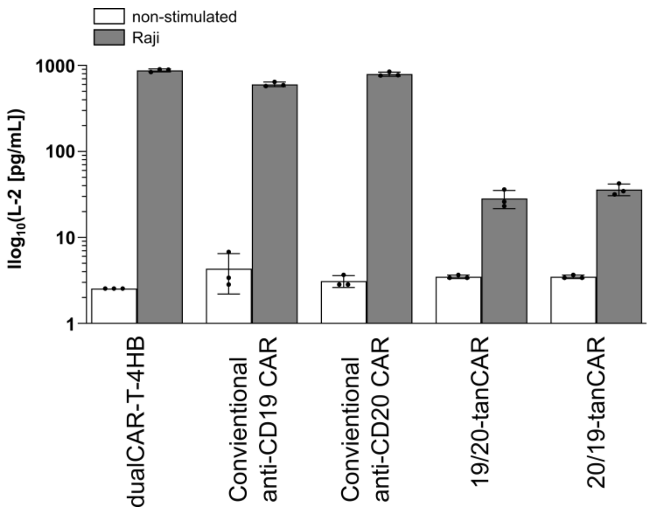

Supplementary Fig. 11. Comparison of dualCAR-T-4HB to conventional and tandem CAR constructs. Analysis of IL-2 production of dualCAR-T-4HB, 2nd generation antiCD19-CAR and antiCD20-CAR, and tandem CARs: 19/20-tanCAR; 20/19-tanCAR introduced into Jurkat cells after stimulation with Raji cells. Values are the mean of three biological replicates ± (s.d.) and representative of one experiment.

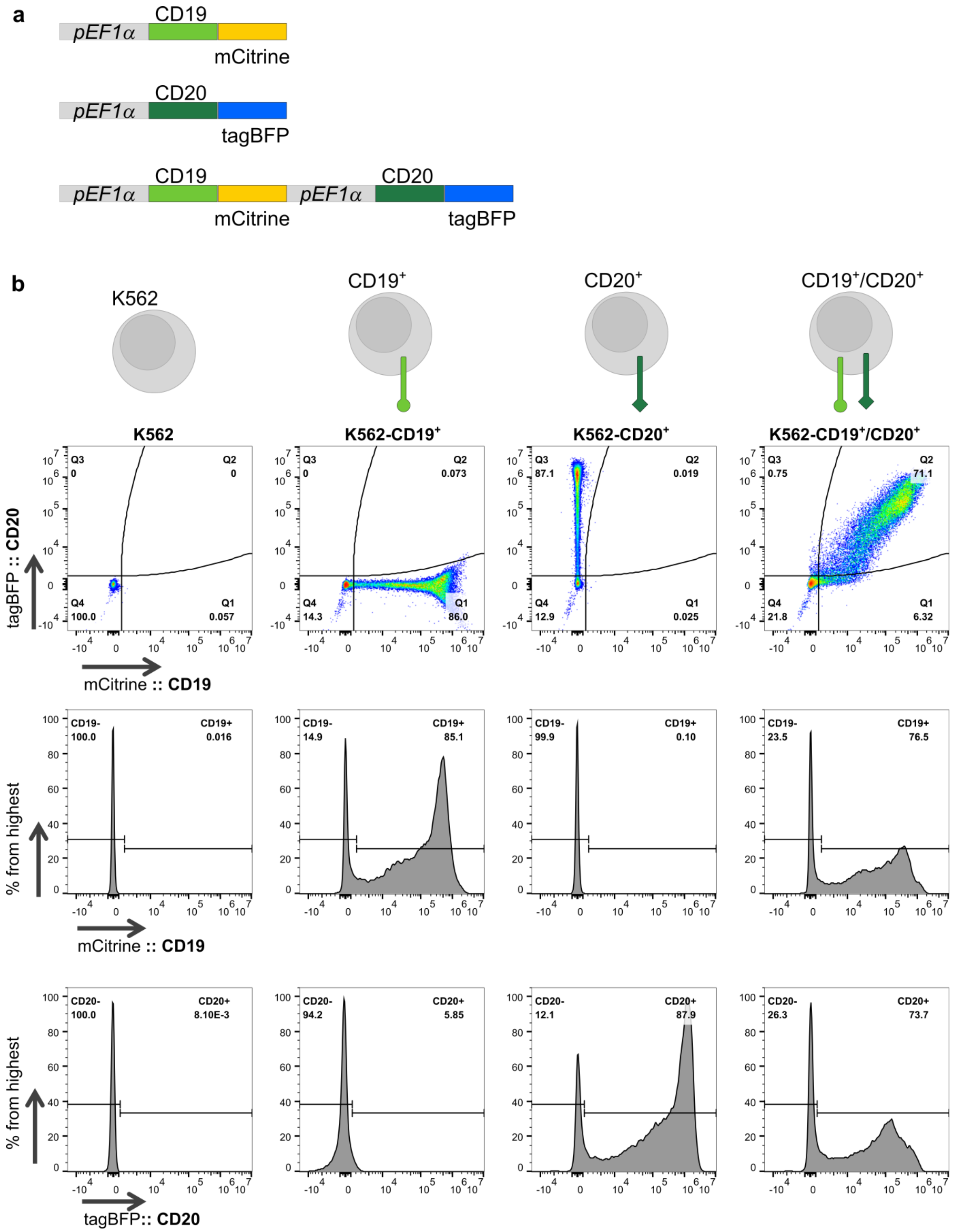

Supplementary Fig. 12. Expression of CD19 and CD20 antigens on K562 electroporated cells. a, Schematic presentation of plasmid constructs for expression of CD19-mCitrine and/or CD20-tagBFP. **b**, Indirect detection of CD19 and CD20 antigen expression was carried out by measurement of mCitrine and tagBFP, respectively, by flow cytometry. Generated K562-CD19+, K562-CD20+ and

K562-CD19+/CD20+ cells were compared to starting K562 cell line. The percentages of positive CD19+, CD20+ or CD19+/CD20+ cells was used for calculation of needed cells for co-culturing experiments.
