# Supplementary material for: Segmentation strategy of de novo designed four-helical bundles expands protein oligomerization modalities for cell regulation
Source: Nat Commun. 2023 Apr 8;14:1995. doi: 10.1038/s41467-023-37765-6 (PMC10082849; doi:10.1038/s41467-023-37765-6)
Supplement: Supplementary file 2 — Reporting Summary [file 41467_2023_37765_MOESM2_ESM.pdf]

nature portfolio

Corresponding author(s): prof. dr. Roman Jerala

Last updated by author(s): Mar 28, 2023

Reporting Summary

Nature Portfolio wishes to improve the reproducibility of the work that we publish. This form provides structure for consistency and transparency in reporting. For further information on Nature Portfolio policies, see our Editorial Policies and the Editorial Policy Checklist.

Statistics

For all statistical analyses, confirm that the following items are present in the figure legend, table legend, main text, or Methods section.

n/a	Confirmed	
[ ]	[x]	The exact sample size (*n*) for each experimental group/condition, given as a discrete number and unit of measurement
[ ]	[x]	A statement on whether measurements were taken from distinct samples or whether the same sample was measured repeatedly
[ ]	[x]	The statistical test(s) used AND whether they are one- or two-sided<br>*Only common tests should be described solely by name; describe more complex techniques in the Methods section.*
[x]	[ ]	A description of all covariates tested
[x]	[ ]	A description of any assumptions or corrections, such as tests of normality and adjustment for multiple comparisons
[ ]	[x]	A full description of the statistical parameters including central tendency (e.g. means) or other basic estimates (e.g. regression coefficient) AND variation (e.g. standard deviation) or associated estimates of uncertainty (e.g. confidence intervals)
[ ]	[x]	For null hypothesis testing, the test statistic (e.g. *F*, *t*, *r*) with confidence intervals, effect sizes, degrees of freedom and *P* value noted<br>*Give P values as exact values whenever suitable.*
[x]	[ ]	For Bayesian analysis, information on the choice of priors and Markov chain Monte Carlo settings
[x]	[ ]	For hierarchical and complex designs, identification of the appropriate level for tests and full reporting of outcomes
[x]	[ ]	Estimates of effect sizes (e.g. Cohen's *d*, Pearson's *r*), indicating how they were calculated

Our web collection on statistics for biologists contains articles on many of the points above.

Software and code

Policy information about availability of computer code

Data collection	BioTek's Synergy™ Mx Microplate Reader and Gen5 Microplate Data Collection & Analysis software was used to measure absorbance, Orion II Microplate Luminometer, Mithras LB 940 or Centro LB 963 microplate readers from BERTHOLD TECHNOLOGIES and Simplicity 4.2 software, were used to measure luminescence, Cytek® Aurora 3L using SpectroFlo 10.8.1 software were used for flow cytometry determination of expression levels of fluorescent proteins and CAR constructs.<br>Molecular models were generating using AlphaFold2. Western blot membrane was imaged with G:Box Chemi XT 4 Chemiluminescence and Fluorescence Imaging System (Syngene).
Data analysis	Graphs and statistical analysis: GraphPad Prism 8.4.3 (http://www.graphpad.com/); Flow cytometry analysis: FlowJo_v10.8.1 (https://www.flowjo.com/solutions/flowjo); Schemes were prepared with Inkscape™ 1.0.2.0, Brooklyn, NY

For manuscripts utilizing custom algorithms or software that are central to the research but not yet described in published literature, software must be made available to editors and reviewers. We strongly encourage code deposition in a community repository (e.g. GitHub). See the Nature Portfolio guidelines for submitting code & software for further information.

Data

Policy information about availability of data

All manuscripts must include a data availability statement. This statement should provide the following information, where applicable:

- Accession codes, unique identifiers, or web links for publicly available datasets
- A description of any restrictions on data availability
- For clinical datasets or third party data, please ensure that the statement adheres to our policy

The authors declare that the data generated in this study and supporting the findings of this study are available in the paper and its supplementary information files. Raw data are available from the corresponding author upon reasonable request. The original 4HB design structures for some 4HBs used in this study are available in protein data bank (PDB) database under following accession numbers: DHD13_XAAA (PDB:6DMP); DHD9 (PDB: 5J7). Source data are provided with this paper.

Human research participants

Policy information about studies involving human research participants and Sex and Gender in Research.

Reporting on sex and gender	N/A
Population characteristics	N/A
Recruitment	N/A
Ethics oversight	N/A

Note that full information on the approval of the study protocol must also be provided in the manuscript.

Field-specific reporting

Please select the one below that is the best fit for your research. If you are not sure, read the appropriate sections before making your selection.

[x] Life sciences [ ] Behavioural & social sciences [ ] Ecological, evolutionary & environmental sciences

For a reference copy of the document with all sections, see nature.com/documents/nr-reporting-summary-flat.pdf

Life sciences study design

All studies must disclose on these points even when the disclosure is negative.

Sample size	At least three independent replicates were taken for each data point in an independent experiment. We chose sufficient sample size to establish that the values compared were derived from normal distributions. The number of replicates are denoted in the figure descriptions. No statistical method was used to determine sample size.
Data exclusions	No data were excluded.
Replication	At least two independent experiments were performed with a comparable outcome. The number of independent experiments are denoted in the figure descriptions.
Randomization	No randomization was applied. The replicates from the data points in each group were positioned next to each other on a sample plate.
Blinding	No animal or human research participants were used, therefore there was no reason for blinding. Furthermore, all measurements in independent experiment were performed at the same time with the identical settings.

Reporting for specific materials, systems and methods

We require information from authors about some types of materials, experimental systems and methods used in many studies. Here, indicate whether each material, system or method listed is relevant to your study. If you are not sure if a list item applies to your research, read the appropriate section before selecting a response.

Materials & experimental systems

n/a	Involved in the study	
☐	☒	Antibodies
☐	☒	Eukaryotic cell lines
☒	☐	Palaeontology and archaeology
☒	☐	Animals and other organisms
☒	☐	Clinical data
☒	☐	Dual use research of concern

Methods

n/a	Involved in the study	
☒	☐	ChIP-seq
☐	☒	Flow cytometry
☒	☐	MRI-based neuroimaging

Antibodies

Antibodies used	Anti-c-Myc antibody produced in rabbit (Sigma-Aldrich®, C3956);<br>Anti-Hsp70 antibody [N27F3-4] (Abcam, ab47454) - Mouse monoclonal [N27F3-4] to Hsp70;<br>Goat Anti-Rabbit IgG H&L (HRP) (Abcam, ab6721);<br>Peroxidase AffiniPure Goat Anti-Mouse IgG (H+L) (© Jackson ImmunoResearch Europe Ltd., 115-035-003);<br>Alexa Fluor® 647 anti-c-Myc Antibody (© BioLegend, Inc., 626810)
Validation	https://www.sigmaaldrich.com/SI/en/product/sigma/c3956<br>https://www.abcam.com/products/primary-antibodies/hsp70-antibody-n27f3-4-ab47454.html<br>https://www.abcam.com/products/secondary-antibodies/goat-rabbit-igg-hl-hrp-ab6721.html<br>https://www.jacksonimmuno.com/catalog/products/115-035-003<br>https://www.biolegend.com/en-ie/products/alexa-fluor-647-anti-c-myc-antibody-20210

Eukaryotic cell lines

Policy information about cell lines and Sex and Gender in Research

Cell line source(s)	From ATCC culture collection: HEK293T cells (CRL-11268); Jurkat E6.1 cells (TIB-152); K-562 cells (CCL-243), Raji (CCL-86)
Authentication	Provided by ATCC culture collection. Cell lines were authenticated based on morphology comparison with ATCC culture collection database images.
Mycoplasma contamination	negative for mycoplasma - guaranteed from the supplier
Commonly misidentified lines<br>(See ICLAC register)	No commonly misidentified cell lines were used in this study.

Flow Cytometry

Plots

Confirm that:

☒ The axis labels state the marker and fluorochrome used (e.g. CD4-FITC).

☒ The axis scales are clearly visible. Include numbers along axes only for bottom left plot of group (a 'group' is an analysis of identical markers).

☒ All plots are contour plots with outliers or pseudocolor plots.

☒ A numerical value for number of cells or percentage (with statistics) is provided.

Methodology

Sample preparation	For CD19 and CD20 expression on K562 cells: cells were washed twice with buffer (10% BSA in PBS) and expression of genetically fused fluorescent proteins was analyzed on a Cytek® Aurora running SpectroFlo software. Flow cytometry data were analyzed with FlowJo v 10.8.1 software.<br>For CAR expression on Jurkat cells: 5×105 cells were collected 48h post electroporation and centrifuged for 5 min at 250 g. Pellet was washed with 1 mL PBS supplemented with 10% FBS and cnetrintrifugated for 5 min at 250 g. Pellet was resuspended in 80 uL FcR blocking reagent for 10 min at 4 °C. Antibodies against Myc-tag labeled with Alexa Fluor 647 were added in the ratio 1:100 for 30 min at 4 °C. After incubation cells were washed with addition of 1,8 mL PBS supplemented with 10% FBS and cnetrintrifugated for 5 min at 250 g. Pellet was resuspended in 300 uL PBS supplemented with 10% FBS. Fluorescence was detected with Cytek® Aurora spectral flow cytometer. Flow cytometry data were analyzed with FlowJo v 10.8.1 software.
Instrument	Cytek® Aurora 3L (Cytek® Biosciences)
Software	SpectroFlo version 2.2.0.2

Cell population abundance	We did not perform flow cytometry sorting (FACS) experiments. Our results present whole sample analysis of either fluorescent protein expression, protein of interest fused to fluorescent protein or labeling with antibodies. Gating strategy of the whole sample was performed to determine cell population gates and fluorochrome-positive cells.
Gating strategy	Cells were gated on singlets (according to FSC-A on FSC-H). FSC-A/FSC-H were used to determine cell population gates.

☒ Tick this box to confirm that a figure exemplifying the gating strategy is provided in the Supplementary Information.
