# Supplementary material for: Segmentation strategy of de novo designed four-helical bundles expands protein oligomerization modalities for cell regulation
Source: Nat Commun. 2023 Apr 8;14:1995. doi: 10.1038/s41467-023-37765-6 (PMC10082849; doi:10.1038/s41467-023-37765-6)
Supplement: Supplementary file 4 — Description of Additional Supplementary Files [file 41467_2023_37765_MOESM4_ESM.pdf]

Title: Supplementary Data 1:
Description: A list and sequences of all constructs used or designed in this study. Amino acid or DNA sequences for all used constructs in the manuscript are divided into topical groups with function-annotated parts of the sequences.
